# Multiplex metagenomic sequencing for rapid viral pathogen identification and surveillance in clinical specimens

**DOI:** 10.1186/s12879-025-11952-w

**Published:** 2025-11-10

**Authors:** Yu-Siang Su, Wei-Hsiang Tsai, Han-Chieh Wu, Yueh-Tzu Chiu, Ni-Rong Jiang, Chien-Yu Lee, Shu-Hsing Cheng, Chih-Ting Huang, Chia-Yu Chi, En-Ju Lin, Yi-Ping Kuo, Wan-Ting Tsai, Chih-Feng Tien, Yu-Chieh Liao, Kuan-Lin Lee, Feng-Jui Chen, Guann-Yi Yu

**Affiliations:** 1 National Institute of Infectious Diseases and Vaccinology, National Health Research Institutes, 35 Keyan Road, Zhunan, 35053 Taiwan; 2 Department of Infectious Diseases, Taoyuan General Hospital, Ministry of Health and Welfare, Taoyuan, Taiwan; 3 Department of Pediatrics, Taoyuan General Hospital, Ministry of Health and Welfare, Taoyuan, Taiwan; 4 School of Public Health, College of Public Health, Taipei Medical University, New Taipei, Taiwan; 5 Institute of Population Health Sciences, National Health Research Institutes, Zhunan, Taiwan; 6 Department of Biological Science and Technology, National Yang Ming Chiao Tung University, Hsinchu, Taiwan

**Keywords:** Nanopore sequencing, Surveillance, Adenovirus, Respiratory syncytial virus, Human parainfluenza virus, Sapporovirus

## Abstract

**Background:**

Rapid and accurate viral detection is essential for clinical diagnosis and effective outbreak surveillance. Traditional methods, including culture-based isolation and antigen tests, are time-consuming and limited by tissue tropism. Multiplex PCR panels, although faster, are constrained by predefined targets, limiting their ability to detect novel or unexpected viral strains.

**Methods:**

We applied Oxford Nanopore Technology sequencing (ONT-Seq), a long-read, real-time, and multiplex metagenomic platform, to 85 clinical specimens using a sequence-independent, single-primer amplification (SISPA) workflow. Sequencing results were compared with routine clinical diagnostics for concordance and for identification of co-infections

**Results:**

ONT-Seq achieved 80% concordance with clinical diagnostics and identified co-infections in 7% of cases missed by routine testing, including influenza C virus (ICV), and Sapporovirus. Among 58 adenovirus-positive cases, 31 samples with over 80% genome coverage at 20× depth were used for phylogenetic analysis, revealing adenovirus B3 as the predominant circulating strain.

**Conclusions:**

ONT-based metagenomic sequencing enhances the detection of both known and emerging viruses in clinical specimens. Its ability to provide real-time, unbiased data supports its utility in improving diagnostic accuracy and viral surveillance.

**Clinical Trial:**

Not applicable.

**Supplementary information:**

The online version contains supplementary material available at 10.1186/s12879-025-11952-w.

## Abstract (pa)

Accurately identifying viruses is essential for diagnosing infections and tracking outbreaks. Traditional methods, such as growing viruses in the lab or using rapid antigen tests, can be slow and may not detect all viruses. PCR tests improve detection but can only identify specific, known viruses. In this study, we used Oxford Nanopore sequencing (ONT-Seq), a real-time genetic sequencing technology, to analyze 85 patient samples. Our approach correctly identified viruses in 80% of cases compared to standard hospital tests and even detected co-infections in 7% of cases that routine tests missed. This sequencing method also allowed us to determine the exact virus type, helping track how viruses spread. Our findings show that metagenomic sequencing is a powerful tool for identifying a wide range of viruses quickly and accurately, improving both diagnosis and outbreak monitoring.

## Background

Emerging and reemerging viral outbreaks, such as SARS virus [1], SARS-CoV-2 [2], Zika virus [3], and monkeypox virus [4], have posed significant public health challenges. Rapid and accurate diagnosis is crucial for effective response and containment. Current diagnostic approaches involve detecting live viruses, viral nucleic acids, antigens, or virus-specific antibodies in clinical specimens [5]. Virus isolation through cell culture has historically played an important role in diagnosing infections and characterizing viruses, although its use in routine clinical diagnostics has markedly declined in recent decades. This method requires specific cell lines or primary tissue cultures and can take days to weeks. Some viruses, such as Hepatitis B and C, Human Papillomavirus, and Norovirus, are particularly difficult to culture. Although virus culture is now mainly used in specialized or reference laboratories, it is still occasionally applied as a supplementary approach in certain clinical centers. Consequently, nucleic acid detection methods such as polymerase chain reaction (PCR), multiplex assays (e.g., film arrays), rapid antigen tests, and serological assays have become the mainstay of clinical diagnostics in many countries. The selection of appropriate detection methods relies heavily on experienced clinicians. However, newly emerging viral pathogens may evade established detection techniques, delaying identification. In such cases, agnostic diagnostic approaches could provide a crucial solution in clinical settings [6].

Advancements in next-generation sequencing (NGS) have driven the application of metagenomic NGS (mNGS) for pathogen diagnosis that enable unbiased pathogen detection without preselected targets [6, 7]. Among the available methods, Illumina-based sequencing and Oxford Nanopore Technology sequencing (ONT-seq) are the two primary approaches for mNGS. Illumina-based sequencing employs sequencing-by-synthesis, using fluorescently labeled nucleotides to detect base incorporation [8]. This method generates short, highly accurate reads, making it ideal for applications requiring precise base calling. It also has been widely applied in viral pathogen detection [9–11]. In contrast, ONT-seq utilizes nanopores to analyze single-stranded DNA by measuring electrical current changes as nucleotides pass through [12]. This technology produces long reads, which are particularly advantageous for resolving complex genomic regions and assembling complete genomes [13]. ONT-seq has also been successfully used for viral pathogen identification [14–16]. Recent advancements in rapid barcoding techniques have significantly improved ONT-seq efficiency, allowing multiplex sequencing of up to 96 samples on a single flow cell, substantially reducing per-sample costs and making large-scale applications more affordable [17]. In clinical settings, its real-time data generation capability enables rapid pathogen identification, offering a key advantage in diagnosing acute viral infections.

In this study, ONT-seq was applied to a large-scale analysis of clinical samples to evaluate its accuracy in metagenomic virus detection. A total of 85 clinical samples from Taoyuan General Hospital in Taiwan were analyzed. Sequencing results showed that ONT-seq achieved approximately 80% concordance with clinical diagnostics, and additionally identified pathogens in about 7% of cases. Notably, sequencing data from pathogens with high genome coverage could be directly used for phylogenetic analysis, underscoring the potential of this approach for both rapid viral diagnosis and long-term epidemiological surveillance.

## Materials and methods

### Specimen collection and ethics statement

Clinical specimens from patients suspected of viral infections (excluding COVID-19) were collected at Taoyuan General Hospital (TYGH), Taoyuan, Taiwan. The samples included feces, cerebrospinal fluid, tissues, and respiratory specimens (e.g., sputum, nasopharyngeal aspirates) with relevant metadata, including infection type, age group, patient ward type (outpatient, emergency, general ward, or intensive care unit), sample type, hospital admission date, sampling date, sex, and admission reason. The clinical laboratory diagnostic records of viral infections were also collected. Ethical approval for all aspects of the study—including specimen collection, temporary storage, metagenomic sequencing–based pathogen detection, and viral isolation—was obtained from the Institutional Review Boards of Taoyuan General Hospital (TYGH112017) and the National Health Research Institutes (EC1110901-E). This manuscript reports only the pathogen detection and sequencing components of the approved protocol. All procedures were conducted in accordance with the Declaration of Helsinki and applicable ethical guidelines.

### Integrated diagnostic and experimental workflow

**Fig. 1:**
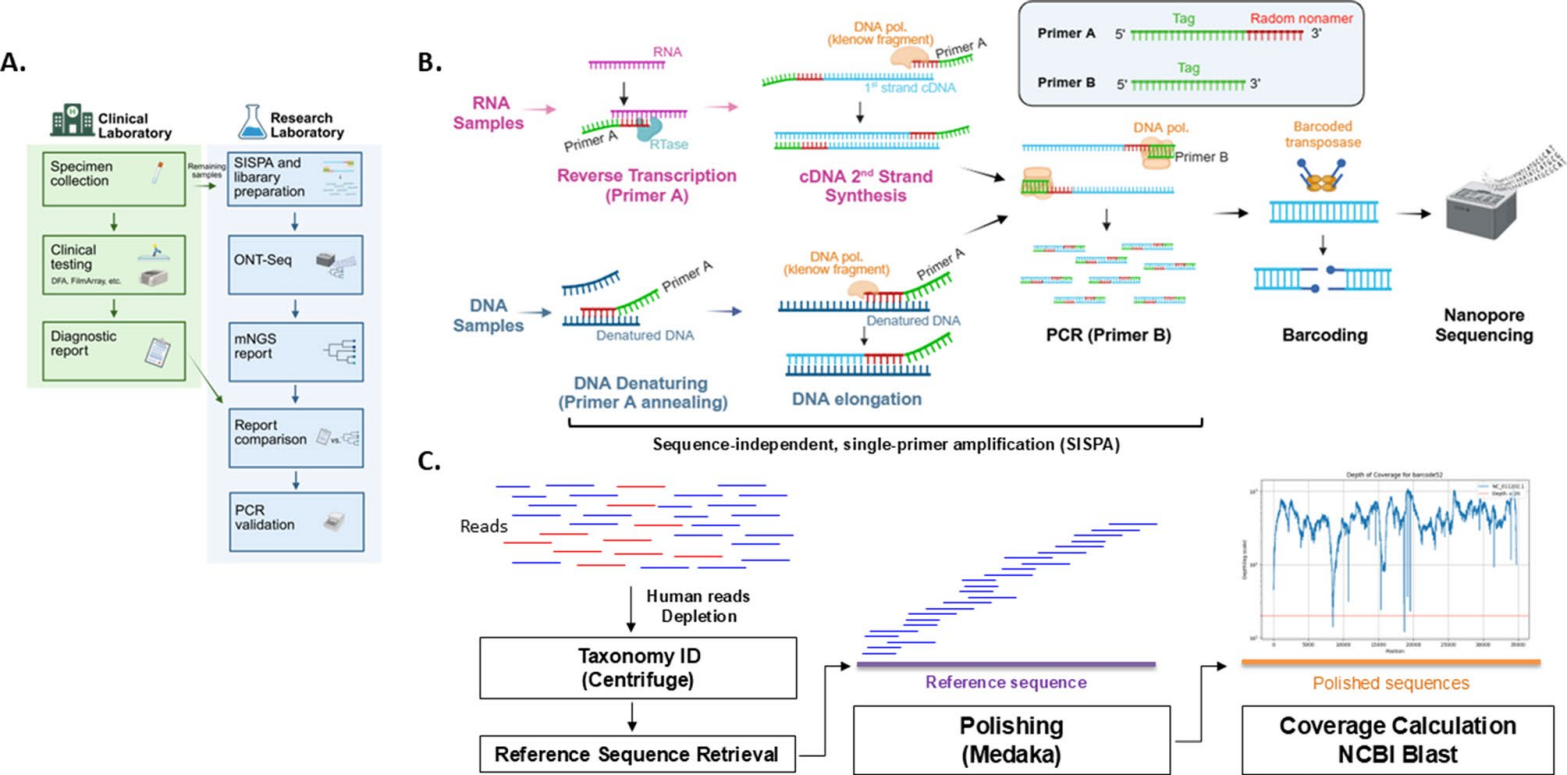
Workflow and sequencing data analysis pipeline. (**A**) overview of the diagnostic workflow for clinical specimens in the clinical laboratory and the research laboratory, showing how residual clinical samples were processed for ONT-based metagenomic sequencing and PCR validation. (**B**) illustration for the sequence-independent, single-primer amplification (SISPA) workflow tailored for Oxford Nanopore technology sequencing (ONT-seq). (**C**) pipeline for viral identification and genome assembly. (**A**) and (**B**) were created with BioRender

The clinical and research laboratories operated independently in this study (Fig. 1A). Clinical diagnostic testing for viral infections was conducted at Taoyuan General Hospital using the D3 Ultra 8™ DFA Respiratory Virus Screening & Identification Kit (Diagnostic Hybrids Inc.) and the BioFire FilmArray Panels (bioMérieux). Residual clinical specimens were then processed in the research laboratory, where they underwent sequence-independent single-primer amplification (SISPA) followed by Oxford Nanopore Technologies-based metagenomic next-generation sequencing (ONT-mNGS). In addition, all 85 specimens included in this study were analyzed using target-specific conventional PCR assays in the research laboratory to validate the mNGS findings. The PCR results for all specimens are summarized in Table S1.

### Nanopore sequencing workflow overview

A comprehensive metagenomic workflow was established for unbiased viral pathogen detection using sequence-independent, single-primer amplification (SISPA) [18, 19] and Oxford Nanopore sequencing (Fig. 1A–C). Patient specimens were first resuspended in Hanks’ Balanced Salt Solution (HBSS), filtered through 0.22 µm filters to remove host cells and debris, and treated with DNase to degrade residual host genomic DNA. Viral RNA and DNA were then separately extracted and subjected to SISPA: RNA samples were reverse-transcribed using SISPA primer A (tagged random nonamer), followed by second-strand synthesis and PCR amplification with primer B (tag only); DNA samples were processed in parallel with the same primer set. The resulting amplicons were barcoded using the ONT transposase-based rapid barcoding kit and sequenced on the MinION platform. Basecalled reads were processed through a bioinformatics pipeline including human-read depletion, taxonomic classification with Centrifuge, reference-based polishing with Medaka, and genome coverage/depth calculation with NCBI BLAST. The detailed experimental procedures are described in the following subsections.

### Viral nucleic acid preparation

Clinical samples were adjusted to a final volume of 500 µL using Hanks’ Balanced Salt Solution (HBSS) and filtered through a 0.22 µm centrifuge tube filter (Costar) to remove most host cells and sample debris. A total of 445 µL of the filtered sample was then mixed with 50 µL of 10X TURBO DNase™ Reaction Buffer and 5 µL of TURBO DNase™ (2 U/µL, Invitrogen), yielding a final reaction volume of 500 µL. The mixture was incubated at 37 °C for 30 minutes in a dry bath to eliminate residual genomic DNA. Following DNase treatment, 200 µL and 280 µL of the processed sample were used for viral DNA and RNA extraction, respectively, using the QIAamp^®^ DNA Mini Kit (QIAGEN) and QIAamp^®^ Viral RNA Mini Kit (QIAGEN), following the manufacturer’s instructions. To enhance nucleic acid precipitation efficiency, linear polyacrylamide (50 µg/mL) was added at 1% (v/v) of the lysis buffer during DNA and RNA extraction. Extracted RNA was further treated with TURBO DNase at 37 °C for 30 minutes and purified using the RNeasy^®^ MinElute^®^ Cleanup Kit (QIAGEN).

### Sequence-independent, single-primer amplification (SISPA)

RNA samples preparation was performed as previously described with minor modifications [14, 20], Briefly, 4 µL of purified RNA was mixed with 1 µL of SISPA primer A (40 pmol/µL, 5’-GTTTCCCACTGGAGGATA-(N9)-3’) and subjected to reverse transcription (RT) using the SuperScript™ IV First-Strand cDNA Synthesis System (Invitrogen). Second-strand cDNA synthesis was subsequently performed using Sequenase Version 2.0 DNA Polymerase (Applied Biosystems™). A 5-µL reaction mixture containing 1 µL of 5X Sequenase buffer, 3.8 µL of ddH₂O, and 0.15 µL of Sequenase was directly added to the RT reaction, followed by incubation at 37 °C for 8 min. An additional Sequenase mixture, consisting of 0.45 µL of Sequenase dilution buffer and 0.15 µL of Sequenase, was then added to the reaction, followed by a second incubation at 37 °C for 8 min. Two units of RNaseH were added to the reaction mixture and incubated at 37 °C for 20 min before further processing.

For DNA sample preparation, 9 µL of extracted DNA was mixed with 1 µL of SISPA primer A (40 pmol/µL, 5’-GTTTCCCACTGGAGGATA-(N9)-3’) and incubated at 95 °C for 5 min, 65 °C for 10 min, and subsequently cooled on ice for 5 min to allow primer A annealing. The DNA extension step was performed by adding 5 µL of Sequenase reaction mixture, consisting of 1 µL of 5X Sequenase buffer, 1 µL of 10 mM dNTP, 2.8 µL of ddH₂O, and 0.15 µL of Sequenase, followed by incubation at 37 °C for 8 min. An additional Sequenase mixture, containing 0.45 µL of Sequenase dilution buffer and 0.15 µL of Sequenase, was then added to the reaction, followed by a second incubation at 37 °C for 8 min. The reaction was then stored on ice until further processing.

For the PCR amplification step, cDNA or DNA products processed with SISPA primer A were further amplified by PCR using AccuTaq™ LA DNA Polymerase (Sigma-Aldrich) and SISPA primer B (100 pmol/µL, 5’-GTTTCCCACTGGAGGATA-3’). The PCR mixture per reaction, consisting of 5 µL of primer A-processed products, 5 µL of AccuTaq LA 10X Buffer, 2.5 µL of dNTP mix, 1 µL of DMSO, 0.5 µL of AccuTaq LA DNA Polymerase, 35 µL of nuclease-free water, and 1 µL of Primer B, was performed as follows: 98 °C for 30 s, followed by 30 cycles of 94 °C for 15 s, 50 °C for 20 s, and 68 °C for 2 min, followed by a final extension at 68 °C for 10 min and a hold at 4 °C. The final PCR products were purified using the QIAquick PCR Purification Kit (QIAGEN) following the manufacturer’s instructions and eluted in 30 µL of ddH₂O for downstream library preparation.

### Library preparation and MinION sequencing

The DNA concentration was measured using a Qubit fluorometer. Sequencing libraries were constructed following the manufacturer’s protocol for the Rapid Barcoding Kit 96 V14 (SQK-RBK114.96). For each patient specimen, 50 ng of SISPA-amplified DNA derived from both DNA and RNA preparations was separately combined with 1 µL of a unique barcode. Each preparation was then adjusted to a total volume of 10 µL using nuclease-free water. For samples with an input mass of less than 50 ng due to low concentration, 9 µL of DNA was used. The library was loaded onto a GridION SpotON Flow Cell (FLO-MIN114) R10.4.1 for sequencing. MinKNOW v23.04.6 was used for live basecalling in high-accuracy mode with adaptive sampling depletion enabled for all channels, using the human genome assembly Build 38 (https://www.ncbi.nlm.nih.gov/assembly/GCF_000001405.26/) as the reference sequence.

### Sequence data analysis

Host reads were removed by mapping the sequencing reads to the same reference sequence used for adaptive sampling, utilizing Minimap2 (2.28-r1209), Samtools (v1.19.2) and Seqkit (v2.8.2). The non-host pathogen sequencing data was analyzed using Centrifuge software (version 1.0.4), with the minimum length of partial hits set to 60. A list of taxonomy IDs was generated to identify potential viruses present in the sample. Based on the taxonomy IDs, reference sequences corresponding to the identified taxa were retrieved from the NCBI database. The non-host pathogen sequencing data was then aligned to the reference sequences and polished twice with Medaka (v1.11.3). The polished reference sequences were subsequently realigned with the non-host pathogen sequencing data to calculate genome coverage and depth. Finally, the refined sequences were used to identify the best match using NCBI BLAST (Basic Local Alignment Search Tool). For samples where Centrifuge did not issue a species-level call but a few related reads were suspected, the reads were additionally mapped to reference viral genomes using minimap2 to examine their genomic distribution.

### Phylogenetic tree construction

To construct the adenoviral phylogenetic tree, sequences with at least 80% genome coverage at a minimum depth of 20× were selected for analysis. Regions with sequencing depth below 20× were masked with “-” to minimize potential biases in phylogenetic inference. All processed whole-genome sequences, along with reference adenoviral genomes retrieved from NCBI (Human adenovirus B3 [MK813914.1], Human adenovirus B14 [JN032132.1], Human adenovirus C2 [OR735196.1], and Human adenovirus F41 [ON532821.1]), were aligned using MUltiple Sequence Comparison by Log-Expectation (MUSCLE) in SnapGene 8.0 prior to further analysis. Phylogenetic analysis was performed using the Maximum Likelihood method in MEGA 11, applying the Tamura-Nei model with 500 bootstrap replicates to assess branch support and evolutionary relationships. For the analysis focusing on HAdV-B3, the same procedures and parameters were applied, except that only B3 genomes were included and the tree was rooted with an HAdV-B7 reference genome [AY594255.1]. In addition, following the approach of Duan et al. [21], representative genomes were incorporated to delineate the three major whole-genome clusters of HAdV-B3, including Cluster I (prototype GB, [AY599834.1]), Cluster II (historical U.S. strains: [AY599836.1], [KF268128.1], [KF268131.1]), and Cluster III (contemporary lineages from the 2000s–2020s: [KX384958.1], [DQ099432.4], [MK883603.1], [MK883608.1], [MW013770.1], [MW013773.1)].

### Conventional PCR for virus detection

To confirm the presence of viral pathogens identified by nanopore sequencing in clinical specimens, the detection of viral genomes was analyzed using conventional PCR. Detailed PCR protocols for various viruses are provided in the supplementary methods and Table S2.

## Results

**Optimized ONT-Seq Workflow for Unbiased Clinical Viral Detection.** To assess the clinical utility of ONT sequencing for viral pathogen detection, a sequence-independent single-primer amplification (SISPA)-based metagenomic workflow was implemented, as outlined in Fig. 1. A total of 85 clinical specimens with confirmed viral infections, obtained from Taoyuan General Hospital, were processed and sequenced. As the viral nucleic acid content of each specimen was unknown (i.e., DNA or RNA viruses), samples were divided into two aliquots for separate DNA and RNA extraction, followed by SISPA amplification and ONT sequencing (Fig. 1B). Sequencing was performed across six runs, each comprising approximately 16–54 barcoded libraries—well within the 96-barcode capacity of the Rapid Barcoding Kit. The resulting read compositions—including total reads, host (human) reads, bacterial reads, and viral reads—are summarized in Table S1.

**Fig. 2:**
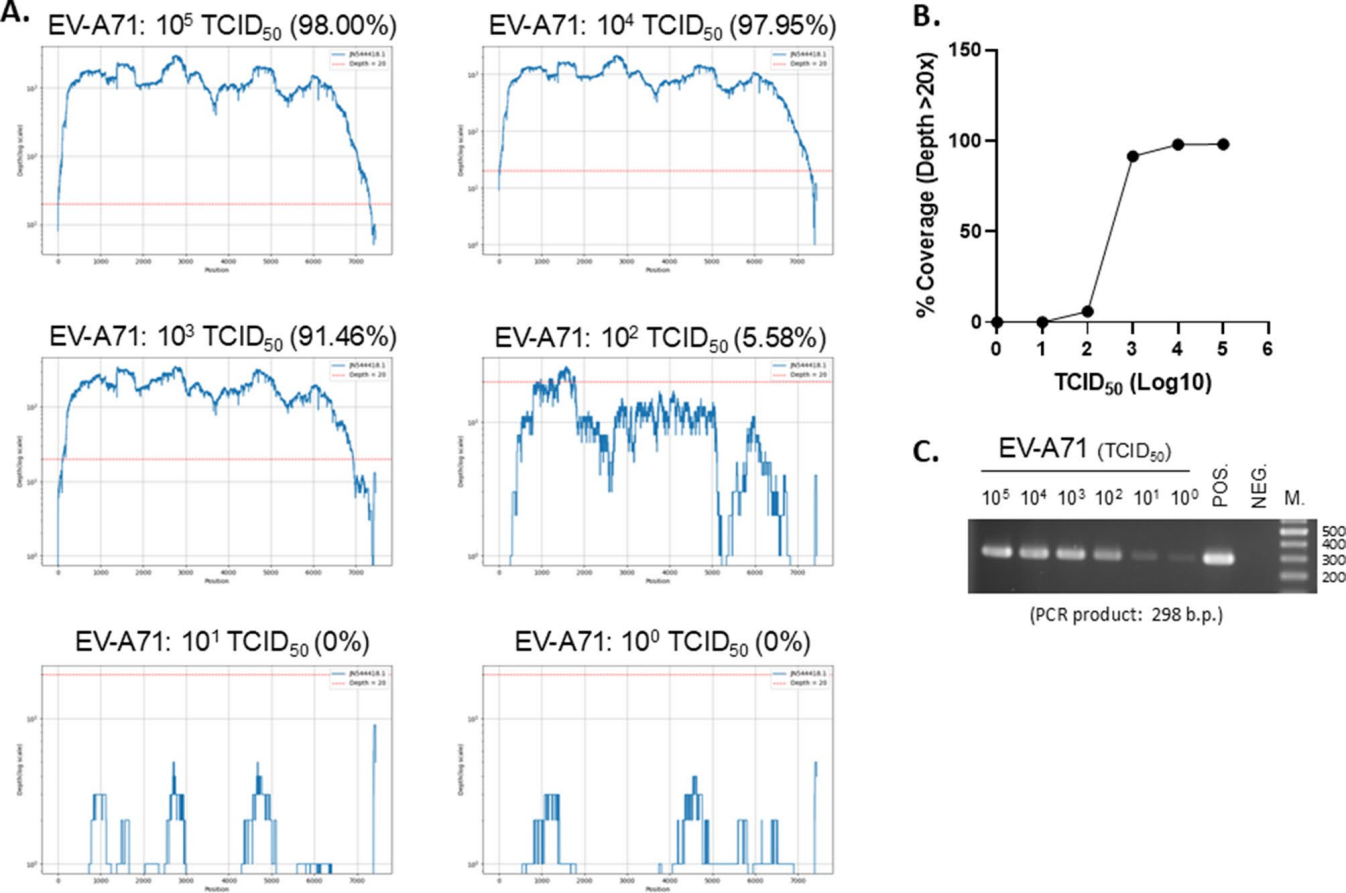
The sensitivity of ONT-seq for RNA viruses was 10^3^ TCID_50_. (**A**) Viral RNA was extracted from different concentrations of Enterovirus A71 and analyzed by ONT-seq. The sequencing read genome coverage plots are shown, with the red line indicating a depth of 20. (**B**) Correlation between viral titer and genome coverage rate. (**C**) Viral RNA from the same samples shown in panel A was reverse-transcribed using random hexamers and subjected to conventional PCR with a Picornaviridae-specific primer set (listed in Table S2), yielding the expected 298 bp amplicon; PCR products were analyzed by 2% agarose gel electrophoresis, with POS. (positive control, known amplifiable cDNA), NEG. (negative control, ddH₂O), and M (DNA ladder)

**ONT-Seq Enables Accurate Viral Detection with Titer Limits.** ONT-seq reads were depleted of human sequences and analyzed using Centrifuge to classify viral taxonomy (Fig. 1B). Reference sequences for the identified viral taxa were retrieved from the NCBI database. Sequencing data, after removal of human sequences, were aligned to these viral reference genomes and polished twice with Medaka. The polished sequences were then realigned with the pathogen sequencing data to assess genome coverage and depth. Finally, refined sequences were analyzed using NCBI BLAST to determine the best match. To assess the accuracy and sensitivity of the workflow, Enterovirus A71 (EV-A71) cultures with known titers (10°–10^5^ TCID₅₀) were subjected to ONT-seq (Fig. 2). Centrifuge analysis identified enterovirus reads in samples with titers ≥10^3^ TCID₅₀, where genome coverage exceeding 20× was over 90%. However, at titers of 1–100 TCID₅₀, Centrifuge’s per-read classifications included a few enterovirus-consistent reads, but under Pavian’s default filters these did not meet the criteria for a reportable call and thus appeared only at higher taxonomic ranks rather than the species level. For comparison, the same EV-A71 RNA samples were also analyzed using conventional PCR with a Picornaviridae-specific primer set. As shown in Fig. 2C, conventional PCR detected EV-A71 down to 10° TCID₅₀, demonstrating higher sensitivity than the SISPA-based NPS method.

**Clinical Sample Collection and Patient Characteristics.** Residual clinical samples from patients suspected of viral infections were collected after routine diagnostic testing at Taoyuan General Hospital between September 7, 2023, and January 10, 2024, to assess the potential of ONT-seq for viral pathogen detection. To prevent SARS-CoV-2 overrepresentation, COVID-19-positive cases were excluded. A total of 85 patient-consented specimens were analyzed (Table 1), with no gender preference and a predominance from pediatric ward patients. The median age was 4 years (range 0–76 years); 83 patients (97.6%) were < 18 years old, and 1 patient (1.2%) was ≥ 18 years old. Associated diseases included acute otitis, sinusitis, tonsillitis, pharyngoconjunctivitis, bronchitis, pneumonia, asthma, encephalitis, gastroenteritis, colitis, and sepsis. Specimen types comprised nasopharyngeal aspirates (67.1%), sputum (30.6%), cerebrospinal fluid (1.2%), and stool (1.2%).

**Table 1:** Clinical characteristics of samples used in the study

Characteristics	Groups	Number of cases	(%)
Gender	Male	44	51.8
	Female	41	48.2
Sample type	Nasopharynx Aspirate	57	67.1
	Sputum	26	30.6
	Cerebrospinal Fluid	1	1.2
	Stool	1	1.2
Age (Years)	0-3	26	30.6
	3-5	33	38.8
	6-11	23	27.1
	12-17	2	2.4
	>18	1	1.2
Sum		85	100.0

**ONT-Seq Enables Broad Viral Detection Across Diverse Clinical Specimens.** A total of 85 clinical specimens were processed and sequenced across six ONT runs, with approximately 16–54 barcoded libraries per run (within the 96-barcode capacity of the kit used). Because the viral nucleic acids present in each sample were unknown (DNA or RNA viruses), each specimen was split into two aliquots for separate DNA and RNA extraction, followed by SISPA amplification and ONT sequencing (Fig. 2B). All specimens were analyzed for potential RNA and DNA pathogens using ONT-seq. Pathogen identification, sequencing read genome coverage (depth > 20×), and the best matching accession numbers from BLAST analysis, and read composition (total, host, bacterial, and viral reads) for all 85 samples are summarized in Table S1. Representative genome coverage and depth plots of four detected viral genomes are provided in Figure S1, as examples of sequencing genome coverage and depth profiles. In the ONT-seq results, adenovirus (Adv) was the most frequently detected virus, identified in 58 samples (67.4%). Respiratory syncytial virus (RSV) was detected in 10 samples (11.6%), followed by human parainfluenza virus (PIV) in 9 samples (10.5%). Influenza A and C viruses were found in 3 samples (3.5%) and human rhinovirus (HRV) was identified in 2 cases (2.3%). Additionally, adeno-associated virus (AAV), enterovirus (EV), coronavirus (CoV), and Sapporovirus (SaV) were each identified in 1 sample (1.2% each) (Table 2).

**Table 2:** Viral pathogens identified in clinical specimens using nanopore sequencing (NPS)

Virus identified by NPS	Number of cases	%
Human Adenovirus (Adv)	58	67.4
Respiratory Syncytial Virus (RSV)	10	11.6
Human parainfluenza virus (PIV)	9	10.5
Influenza virus	3	3.5
Human Rhinovirus (HRV)	2	2.3
Adeno-associated virus (AAV)	1	1.2
Enterovirus (EV)	1	1.2
Coronavirus (CoV)	1	1.2
Sapporovirus (SaV)	1	1.2

**ONT-Seq Shows High Accuracy with Some Low-Genome Coverage Misses.** In this study, clinical diagnosis results obtained through routine diagnostic methods at Taoyuan General Hospital, which include a combination of multiplex PCR panels (e.g., FilmArray Respiratory Panel), rapid antigen tests, and conventional RT-PCR assays depending on the clinical indication and specimen type. The consistency between clinical diagnosis (CD) and ONT-seq results was evaluated (Table 3). Overall, 68 cases (80%) showed concordant results, while 17 cases (20%) had discrepancies. These included 6 cases (7.1%) where ONT-seq detected co-infecting pathogens (CD < ONT-seq), 10 cases (11.8%) where co-infecting pathogens were identified by the hospital (CD > ONT-seq), and 1 case (1.2%) where different pathogens were identified (CD ≠ ONT-seq).

**Table 3:** Comparison of pathogen identification results between nanopore sequencing and clinical diagnosis

Identification Result	Number of Cases (%)	Case Number	Clinical Diagnosis (CD)	Nanopore Sequencing (NPS) (% Coverage, Depth > 20x)	Reference	Research Lab PCR Validation
		8, 16, 24, 25, 27, 32,	Adv			
		33, 37, 40–43, 46, 48-				
	**52 (61.18%)**	53, 55, 56, 58–69, 71-		*	*	*
		77, 80–84, 86, 87, 89,				
**CD = NPS**		90, 92–94				
	**2 (2.35%)**	70, 78	IAV	*	*	*
	**6 (7.06%)**	11, 13, 17, 45, 47, 57	PIV	*	*	*
	**8 (9.41%)**	3, 4, 7, 9, 12, 15, 26,	RSV	*	*	*
		29				
		1	Adv	Adv F41 (99.98%)	ON815882.1	Adv (+)
				SaV GI.1 (91.52%)	LC504312.1	SaV (+)
		5	RSV	RSV A (3.86%)	NC001803.1	RSV (+)
				HRV C28 (94.50%)	OK017915.1	HRV (+)
		10	Adv	Adv C1 (0%)	AC000017.1	Adv (+)
				ICV		ICV (+)
				(Seg 1: 81.65%)	LC720290.1	
				(Seg 2: 82.82%)	LC739932.1	
				(Seg 3: 67.80%)	OK625707.1	
**CD < NPS**	**6 (7.06%)**			(Seg 4: 99.71%) (Seg 5: 99.61%)	LC720285.1 OK625712.1	
				(Seg 6: 99.15%)	KM504282.1	
				(Seg 7: 99.25%)	LC720288.1	
		20	Adv	Adv B3 (100%)	OR487155.1	Adv (+)
				AAV A2 (99.98%)	OP161118.1	AAV (+)
		38	PIV3	PIV 3 (0.43%)	OR728657.1	PIV3 (+)
				Adv C1 (0%)	AC_000017.1	Adv (+)
		39	RSV	RSV A (12.28%)	PP525326.1	RSV (+)
				Adv B11 (0%)	AY163756.1	Adv (+)
		2	PIV 1	-	-	PIV1 (-)
		6	RSV	-	-	RSV (+)
		14	RSV	-	-	RSV (+)
		18	PIV3	-	-	PIV3 (-)
		28	RSV	-	-	RSV (+)
		44	PIV3	-	-	PIV3 (+)
		54	HRV	HRV (0%)	PP194074.1	HRV (+)
**CD > NPS**	**10 (11.76%)**		PIV3			PIV3 (-)
		79	PIV3	PIV 3 (99.64%)	MN145875.1	PIV3 (+)
			HRV			HRV (-)
			EV			EV (-)
		88	CoV HKU1	CoV HKU1 (0%)	LC654448.1	CoV-HKU1 (-)
			Adv			Adv (+)
		91	EV	EV A (61.42%)	NC_001612.1	EV (+)
			PIV2	PIV2 (5.60%)	NC_003443.1	PIV2 (+)
			HRV			HRV (+)
**CDNPS**	**1 (1.18%)**	31	HSV 2	Adv B11 (0%)	AY163756.1	HSV-2 (-) Adv (+)

Adv: Human Adenovirus; AAV: Adeno-associated virus; CoV: Coronavirus; EV: Enterovirus; ICV: HSV: Human Herpes Virus; HRV: Human Rhinovirus; Inflenza C virus; PIV: Human parainfluenza virus; RSV: Respiratory Syncytial Virus; SaV: Sapporovirus

*NPS results, References, and PCR validation results are listed in Table S1

To further investigate the discrepancies, ONT-seq read genome coverage and depth for the identified pathogens were analyzed, and pathogen detection was repeated using conventional PCR (Table 3 and S1). The additional pathogens detected by ONT-seq showed high read genome coverage in cases #1 (SaV, 91.52%), #5 (HRV, 94.50%), #10 (ICV, 7 genome segments, 81.65%–99.71%), and #20 (AAV, 99.98%). Additionally, adenoviruses identified in cases #38, #39, and #31 were confirmed by conventional PCR. These findings suggest that ONT-seq’s pathogen identification accuracy could reach up to 88.2%. Conversely, in the 10 cases where clinical diagnosis identified additional pathogens (CD > ONT-seq), conventional PCR confirmed the presence of viruses in seven cases (#6, 14, 28, 44, 54, 88, and 91), indicating that ONT-seq failed to detect these viruses. The misidentification of these cases may be related to insufficient viral-associated reads and low genome coverage, which could be caused by low viral loads or by ONT-seq reads not aligning with the regions required for taxonomy calling.

**Fig. 3:**
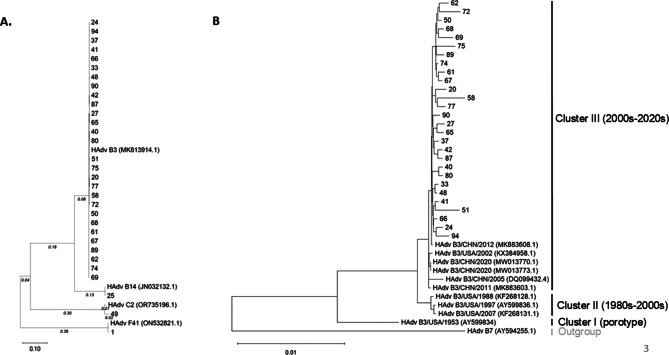
Phylogenetic trees of adenoviruses detected by ONT sequencing.Adenovirus genomes with ≥80% genome coverage at ≥20× depth were analyzed using the maximum likelihood method. (**A**) global whole-genome tree of all qualifying adenovirus genomes (*n* = 31) together with type references (B3, B14, C2, F41). (**B**) HAdV-B3–only whole-genome tree, rooted with HAdV-7, including reference genomes representing three commonly used whole-genome clusters (cluster I: prototype GB; cluster II: historical U.S. strains; cluster III: contemporary lineages from the 2000s–2020s)

**ONT-Seq Provides Accurate Virus Classification for Epidemiological Studies.** More than half of the identified pathogens had over 50% genome coverage (Depth > 20x; Table S1). When viral genomes were identified by ONT-seq with high genome coverage, the assembled genome sequences enabled accurate virus classification by identifying the closest references. Consequently, the sequencing data could further support molecular epidemiology studies. For instance, the Sapporovirus detected in a stool specimen (Case #1) had 91.52% genome coverage and closely matched the GI.1 genotype (LC504312.1). The human rhinovirus identified in Case #5 (94.50% genome coverage) was closely related to the human rhinovirus C28 strain (OK017915.1), while the enterovirus detected in Case #91 (61.42% genome coverage) was closely related to coxsackievirus A16. All 10 RSV-positive cases were classified as subgroup A, with read genome coverage ranging from 3.86% to 83.87%. Based on ONT-seq analysis, the 58 adenovirus-positive specimens were classified into Adv B3, B11, C1, C2, C5, and F41, with genome coverage rates (depth > 20×) ranging from 0% to 99.97%. Adenovirus genome sequences with a genome coverage rate above 80% were further analyzed using the Maximum Likelihood method in MEGA 11: Molecular Evolutionary Genetics Analysis Version 11 (MEGA11) [22]. As shown in Fig. 3, aside from single cases of Adv B14, C2, and F41, Adv B3 was the dominant type, with its genome sequences clustering closely together, suggesting active circulation of Adv B3 serotype in the region. The clinical manifestations associated with Adv B3 infection in patients included sinusitis, tonsillitis, otitis media, pharyngoconjunctivitis, bronchopneumonia, pneumonia, febrile illness, and convulsions. While Adv B14 has been previously linked to severe respiratory diseases with high mortality rates in adults [23], the Adv B14-infected patient in this study presented only with acute sinusitis. Additionally, AdV F41, which is primarily associated with gastroenteritis [24], was identified in a stool sample from a patient diagnosed with gastroenteritis and colitis, who was also co-infected with Sapporovirus.

To further investigate the genetic diversity among AdV B3 cases, a phylogenetic tree was constructed using only AdV B3 genomes along with representative reference strains delineating the three major clusters described by Duan et al. [21], with AdV B7 included as the outgroup (Fig. 3B). Despite all specimens being collected over a relatively short period (September 2023 to January 2024), all AdV B3 genomes were classified within Cluster III. However, they were dispersed across several distinct sub-branches rather than forming a single clonal cluster, suggesting the concurrent circulation of multiple lineages of this serotype during the study period. These findings demonstrate that mNGS data can be directly applied to molecular epidemiological research, providing valuable insights into virus circulation and disease associations.

## Discussion

In this study, we developed and validated a metagenomic sequencing approach using sequence-independent amplification and ONT-Seq for unbiased clinical viral detection. Our analysis successfully identified pathogens in diverse clinical specimens with up to 87% concordance, including co-infection not initially suspected. Notably, more than half of the identified pathogens had genome coverage exceeding 50%, enhancing confidence in pathogen classification. These high-resolution sequencing data provide valuable insights for epidemiological investigations and long-term surveillance. For example, a significant proportion of cases tested positive for adenovirus, and the whole-genome data enabled exploratory phylogenetic analysis. Although all specimens in this study were collected within a five-month period, notable diversity was observed among the AdV-B3 genome sequences. This finding suggests that clinical mNGS data can provide valuable preliminary insights into the genetic diversity of circulating strains. This study highlights the potential of metagenomic sequencing using ONT-Seq combined with SISPA as a practical platform for comprehensive viral pathogen detection, real-time outbreak response, and epidemiological research.

Although viral culture was historically used for diagnosing viral infections, the current standard in hospitals primarily relies on rapid tests, virus-specific PCR, and film array panels. However, not all viral pathogens are culturable, and routine cell lines have limited genome coverage. While PCR and film arrays have significantly improved viral detection, multiple tests are often required. In contrast, unbiased mNGS simplifies the detection process across various specimen types and viruses. Although our sequencing times typically ranged from 24 to 72 hours to maximize output and detection limits, ONT-Seq data can be collected in real time, potentially reducing the time required for pathogen identification. Our findings are aligned with recent work by Charalampous et al. [25], who implemented Nanopore-based metagenomic diagnostics in ICU patients and demonstrated real-world impact on antimicrobial stewardship and decision-making. While their study focused on lower respiratory tract infections in critically ill patients, our proof-of-concept investigation expands the potential of metagenomic sequencing to routine respiratory specimens, including upper respiratory tract infections and outpatient cases. Moreover, our use of SISPA amplification instead of host depletion presents a simplified alternative workflow, albeit with differences in sensitivity and bias profiles that warrant further comparative evaluation.

Despite its high accuracy, ONT-Seq failed to detect certain pathogens in some specimens. In one case, where both EV and HRV were reported by clinical diagnostics, ONT-Seq detected only EV. As EV and HRV belong to the Enterovirus genus within the Picornaviridae family and share high nucleotide sequence identity in conserved regions, distinguishing between them remains challenging. Increased sequencing genome coverage by ONT-seq improved resolution for closely related EV and HRV. Additionally, some cases confirmed by conventional PCR, including RSV (#6, #14, #28), PIV3 (#44), and Adv (#88), were not detected by ONT-Seq. This may be due to low viral genome copies in the specimens, limiting amplification by SISPA, or to amplified genome fragments not aligning with taxonomic classification regions used by Centrifuge. For instance, EV-related reads were present in low-titer EV samples (10°, 10^1^, 10^2^ TCID_50_/ml), but sequencing depth was insufficient for confident identification. Further optimization of the classification process may improve sensitivity without compromising specificity.

Most studies using mNGS for viral pathogen detection have primarily focused on liquid-based clinical samples [11, 26–28], such as plasma, cerebrospinal fluid, and swab samples suspended in viral transport medium. This study included a fecal specimen, which is complex in composition and often challenging for unbiased sequencing. SISPA-ONT-Seq performed robustly, detecting two pathogens with high genome coverage and depth, demonstrating the workflow’s versatility and applicability across diverse specimen types.

While ONT-Seq offers high accuracy in viral pathogen detection, several steps should be streamlined before clinical implementation. Taxonomy identification reports often contain background noise or nonpathogenic viral sequences, requiring expert review to select relevant pathogens. Additionally, DNA and RNA extraction are currently performed separately in the SISPA process to enhance sensitivity. Integrating both nucleic acids into a single automated workflow could improve efficiency, reduce the required specimen volume, shorten processing time, and lower costs, facilitating broader clinical adoption of metagenomic sequencing. As this was a proof-of-concept study, all sequencing and analysis steps were conducted under optimized laboratory conditions and not in a continuous real-time setting. Therefore, exact turnaround time from sample receipt to report generation was not measured.

## Conclusions

This study demonstrates the utility of SISPA combined with ONT-Seq for unbiased metagenomic detection of viral pathogens in clinical specimens. The method showed high concordance with routine diagnostics and identified co-infections missed by standard tests. High genome coverage enabled genotyping and phylogenetic analysis. The workflow performed well across various specimen types, including fecal samples. While challenges remain in sensitivity for low-titer viruses and distinguishing closely related strains, further optimization and workflow integration could support broader clinical adoption. Overall, SISPA-ONT-Seq offers a promising tool for viral diagnosis and surveillance.

## Electronic supplementary material

Below is the link to the electronic supplementary material.

Supplementary Material 1

Supplementary Material 2

Supplementary Material 3

Supplementary Material 4

Supplementary Material 5

## Data Availability

All data generated or analyzed during this study are included in this published article and its supplementary information files.

## Abbreviations

AAV: Adeno-associated virus
Adv: Adenovirus
CD: Clinical diagnosis
cDNA: Complementary DNA
CoV: Coronavirus
dsDNA: Double-stranded DNA
EV: Enterovirus
HRV: Human rhinovirus
ICV: Influenza C virus
IRB: Institutional Review Board
mNGS: Metagenomic next-generation sequencing
ONT-seq: Oxford Nanopore Technology sequencing
PIV: Parainfluenza virus
RSV: Respiratory syncytial virus
SaV: Sapporovirus
SISPA: Sequence-independent, single-primer amplification
TCID₅₀: Tissue culture infectious dose 50%
