# Supplementary material for: Multiplex metagenomic sequencing for rapid viral pathogen identification and surveillance in clinical specimens
Source: BMC Infect Dis. 2025 Nov 10;25:1531. doi: 10.1186/s12879-025-11952-w (PMC12604265; doi:10.1186/s12879-025-11952-w)
Supplement: Supplementary file 1 — Supplementary Material 1 [file 12879_2025_11952_MOESM1_ESM.docx]

**Supplementary Method**

**Conventional PCR validation of mNGS and clinical results**

Conventional PCR assays were performed to validate pathogen identification results from mNGS and clinical diagnostics. DNA extracted from clinical specimens was used directly as a template, while RNA samples were reverse-transcribed into cDNA using the SuperScript™ IV First-Strand Synthesis System (Invitrogen) with random hexamer primers, following the manufacturer’s protocol. Virus-specific primers targeting adenovirus(1), picornaviruses (enterovirus and rhinovirus)(2), rhinovirus(3), coronaviruses(4), herpesviruses (HSV-1, HSV-2, CMV, and EBV)(5), respiratory syncytial virus (RSV) subtypes A and B(6), influenza A virus (LGC Biosearch Technologies), influenza C virus(7), adeno-associated virus(8), and sapporovirus(9) were used. Primer sequences are listed in **Supplementary Table S2**. PCR was performed using 2× Taq DNA Polymerase Master Mix RED (Amplicon) according to the manufacturer’s instructions. Thermal cycling conditions were as follows: initial denaturation at 95°C for 3 min; 40 cycles of 95°C for 30 s, annealing (temperature varied according to primer design or published reference) for 15 s, and extension at 72°C for 30 s; followed by a final extension at 72°C for 10 min.

**References**

1. Sibanda T, Okoh AI. Assessment of the Incidence of Enteric Adenovirus Species and Serotypes in Surface Waters in the Eastern Cape Province of South Africa: Tyume River as a Case Study. The Scientific World Journal. 2012;2012(1):949216.

2. Hyypiä* T, Auvinen P, Maaronen M. Polymerase Chain Reaction for Human Picornaviruses. Journal of General Virology. 1989;70(12):3261-8.

3. Ng KT, Chook JB, Oong XY, Chan YF, Chan KG, Hanafi NS, et al. Performance of a Taqman Assay for Improved Detection and Quantification of Human Rhinovirus Viral Load. Scientific Reports. 2016;6(1):34855.

4. Vijgen L, Moës E, Keyaerts E, Li S, Van Ranst M. A Pancoronavirus RT-PCR Assay for Detection of All Known Coronaviruses. In: Cavanagh D, editor. SARS- and Other Coronaviruses: Laboratory Protocols. Totowa, NJ: Humana Press; 2008. p. 3-12.

5. Zhongliang W, Rui C, Xiangling W, Ding L, Jinrong Z, Yanhai G, et al. Detection of the four major human herpesviruses simultaneously in whole blood and cerebrospinal fluid samples by the fluorescence polarization assay. International Journal of Infectious Diseases. 2010;14(10):e893-e7.

6. Liu W, Chen D, Tan W, Xu D, Qiu S, Zeng Z, et al. Epidemiology and Clinical Presentations of Respiratory Syncytial Virus Subgroups A and B Detected with Multiplex Real-Time PCR. PLOS ONE. 2016;11(10):e0165108.

7. Claas ECJ, Sprenger MJW, Kletera GEM, Beek RV, Quint WGV, Masurela N. Type-specific identification of influenza viruses A, B and C by the polymerase chain reaction. Journal of Virological Methods. 1992;39(1):1-13.

8. Tobiasch E, Burguete T, Klein-Bauernschmitt P, Heilbronn R, Schlehofer JR. Discrimination between different types of human adeno-associated viruses in clinical samples by PCR. Journal of Virological Methods. 1998;71(1):17-25.

9. Oka T, Yamamoto SP, Iritani N, Sato S, Tatsumi C, Mita T, et al. Polymerase chain reaction primer sets for the detection of genetically diverse human sapoviruses. Archives of Virology. 2020;165(10):2335-40.
