# Supplementary material for: Multiplex metagenomic sequencing for rapid viral pathogen identification and surveillance in clinical specimens
Source: BMC Infect Dis. 2025 Nov 10;25:1531. doi: 10.1186/s12879-025-11952-w (PMC12604265; doi:10.1186/s12879-025-11952-w)
Supplement: Supplementary file 2 — Supplementary Material 2 [file 12879_2025_11952_MOESM2_ESM.docx]

**
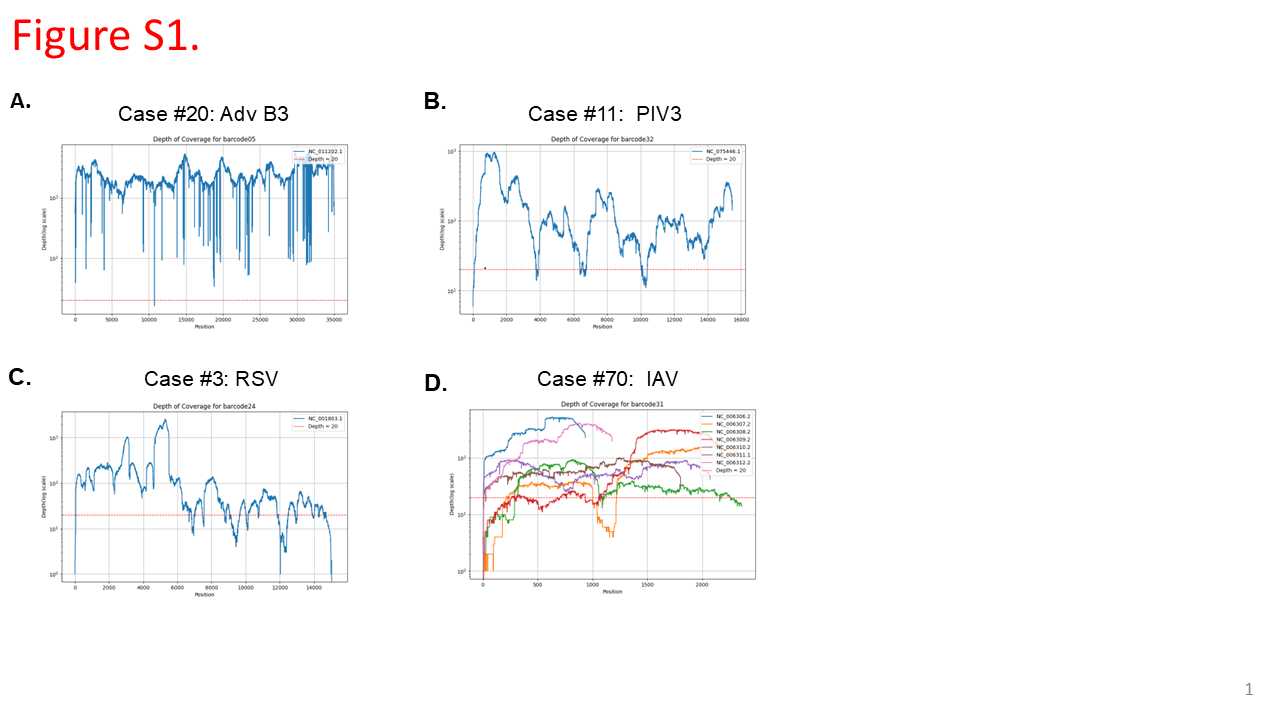
**

**Supplementary Figure Legend**

**Fig. S1 Representative sequencing genome coverage and depth plots for selected viruses detected by SISPA-based ONT-seq.**

(A) Adenovirus B3 (Case #20), (B) Parainfluenza virus 3 (Case #11), (C) Respiratory syncytial virus (Case #3), and (D) Influenza A virus (Case #70). The X-axis indicates the genome position and the Y-axis indicates sequencing depth (log scale). The red dashed line marks the 20× depth threshold used for genome coverage calculation. Reference accession numbers and genome coverage statistics (% genome coverage at depth > 20×) for these cases are provided in Table S1.
