# Supplementary figures and images for: Multiplex metagenomic sequencing for rapid viral pathogen identification and surveillance in clinical specimens
Source: BMC Infect Dis. 2025 Nov 10;25:1531. doi: 10.1186/s12879-025-11952-w (PMC12604265; doi:10.1186/s12879-025-11952-w)

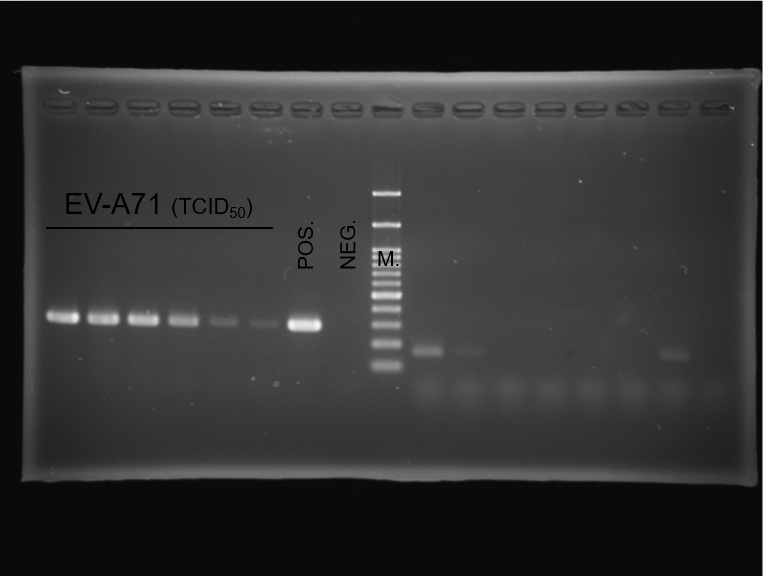

Supplement: Supplementary file 3 — Supplementary Material 3 [file 12879_2025_11952_MOESM3_ESM.tif]
