# Supplementary material for: Multiplex metagenomic sequencing for rapid viral pathogen identification and surveillance in clinical specimens
Source: BMC Infect Dis. 2025 Nov 10;25:1531. doi: 10.1186/s12879-025-11952-w (PMC12604265; doi:10.1186/s12879-025-11952-w)
Supplement: Supplementary file 4 — Supplementary Material 4 [file 12879_2025_11952_MOESM4_ESM.pdf]

Table S1. Summary of data for the 85 clinical samples analyzed in the study.

Case Number	Age	Gender	Specimen Type	Specimen Collection Date	Clinical ID	Clinical Test Method	NGS ID (% Coverage, Depth >20x)	Reference	Research Lab PCR Validation	NGS DNA reaction					NGS RNA reaction				
										Total Reads	Host Reads (%)	Bacteria Reads (%)	Virus Reads (%)	Average Read Length	Total Reads	Host Reads (%)	Bacteria Reads (%)	Virus Reads (%)	Average Read Length
1	1	F	St	9/7/2023	Adv	CLIA	Adv F41 (99.98%)<br>SaV GI.1 (91.52%)	ON815882.1<br>LC504312.1	Adv (+)<br>SaV (+)	304954	299 (0.10%)	79469 (26.06%)	124581 (40.85%)	363.1	63206	71 (0.11%)	39050 (61.78%)	1022 (1.62%)	272
2	0	F	Sp	9/7/2023	PIV1	DFA	–	–	PIV1 (-)	266918	4258 (1.60%)	23317 (8.74%)	511 (0.19%)	287.7	16609	215 (1.29%)	3414 (20.56%)	23 (0.14%)	287.3
3	0	M	Sp	9/8/2023	RSV	DFA	RSV A (83.87%)	PP969976.1	RSV (+)	146990	121173 (82.44%)	2406 (1.64%)	303 (0.21%)	263.9	75605	23555 (31.16%)	16149 (21.36%)	8711 (11.52%)	300
4	0	M	Sp	9/8/2023	RSV	DFA	RSV A (20.03%)	KF826849.1	RSV (+)	104632	25870 (24.72%)	7272 (6.95%)	395 (0.38%)	259.4	19672	5640 (28.67%)	3971 (20.19%)	632 (3.21%)	284.6
5	3	M	Sp	9/8/2023	RSV	DFA	RSV A (3.86%)<br>HRV C28 (94.50%)	NC001803.1<br>OK017915.1	RSV (+)<br>HRV (+)	214244	194558 (90.81%)	801 (0.37%)	19 (0.01%)	185	30114	18352 (60.94%)	2033 (6.75%)	287 (0.95%)	292.6
6	3	F	Sp	9/8/2023	RSV	DFA	–	–	RSV (+)	128632	74664 (58.04%)	4632 (3.60%)	455 (0.35%)	253.7	4484	141 (3.14%)	3151 (70.27%)	2 (0.04%)	282.9
7	2	M	Sp	9/10/2023	RSV	DFA	RSV A (35.21%)	PP151374.1	RSV (+)	230299	176428 (76.61%)	2787 (1.21%)	13847 (6.01%)	273.7	122623	111509 (90.94%)	5792 (4.72%)	2103 (1.72%)	316.2
8	5	M	Sp	9/11/2023	Adv	DFA	Adv B11 (17.63%)	AY598970.1	Adv (+)	110388	95206 (86.25%)	581 (0.53%)	1598 (1.45%)	212.1	8474	721 (8.51%)	6244 (73.68%)	7 (0.08%)	258.9
9	1	F	Sp	9/11/2023	RSV	DFA	RSV A (18.81%)	KF826849.1	RSV (+)	75249	19069 (25.34%)	5540 (7.36%)	63 (0.08%)	270.9	11326	2213 (19.54%)	1076 (9.50%)	405 (3.58%)	294.3
10	3	F	Sp	9/11/2023	Adv	DFA	Adv C1 (0%)<br>ICV<br>(Seg 1: 81.65%)<br>(Seg 2: 82.82%)<br>(Seg 3: 67.80%)<br>(Seg 4: 99.71%)<br>(Seg 5: 99.61%)<br>(Seg 6: 99.15%)<br>(Seg 7: 99.25%)	AC000017.1<br><br>LC720290.1<br>LC739932.1<br>OK625707.1<br>LC720285.1<br>OK625712.1<br>KM504282.1<br>LC720288.1	Adv (+)<br><br>ICV (+)	111880	101301 (90.54%)	298 (0.27%)	72 (0.06%)	210.1	17777	8187 (46.05%)	724 (4.07%)	3541 (19.92%)	315.1
11	2	M	Sp	9/12/2023	PIV 3	DFA	PIV 3 (95.43%)	LC817395.1	PIV3 (+)	28086	2687 (9.57%)	2438 (8.68%)	59 (0.21%)	276.9	17989	4493 (24.98%)	661 (3.67%)	7196 (40.00%)	317.6
12	3	M	Sp	9/10/2023	RSV	DFA	RSV A (4.58%)	MG642056.1	RSV (+)	187113	150190 (80.27%)	1380 (0.74%)	8559 (4.57%)	254.1	56293	50975 (90.55%)	552 (0.98%)	184 (0.33%)	273.7
13	4	M	Sp	9/12/2023	PIV 2	DFA	PIV 2 (1.45%)	NC_003443.1	PIV2 (+)	93245	810 (0.87%)	8554 (9.17%)	164 (0.18%)	255	6489	287 (4.42%)	1005 (15.49%)	41 (0.63%)	274.3
14	2	M	Sp	10/2/2023	RSV	DFA	–	–	RSV (+)	156668	135719 (86.63%)	1074 (0.69%)	97 (0.06%)	217.9	12044	9820 (81.53%)	571 (4.74%)	N/D	314.1
15	1	F	Sp	10/2/2023	RSV	DFA	RSV A (37.94%)	PP969976.1	RSV (+)	130218	109136 (83.81%)	1770 (1.36%)	53 (0.04%)	224.7	49489	14033 (28.36%)	7812 (15.79%)	1948 (3.94%)	285.7
16	5	F	Sp	10/4/2023	Adv	DFA	Adv B3 (19.35%)	MK813914.1	Adv (+)	92848	78633 (84.69%)	841 (0.91%)	1428 (1.54%)	247.4	11420	9102 (79.70%)	443 (3.88%)	4 (0.04%)	312.8
17	0	F	Sp	10/3/2023	PIV 2	DFA	PIV 2 (11.64%)	LC720864.1	PIV2 (+)	130141	15224 (11.70%)	10403 (7.99%)	198 (0.15%)	269.3	10729	2324 (21.66%)	862 (8.03%)	412 (3.84%)	304.6
18	1	F	Sp	11/20/2023	PIV 3	DFA	–	–	PIV3 (-)	513470	182874 (35.62%)	186 (0.04%)	263 (0.04%)	332.3	379774	6452 (1.70%)	1289 (0.34%)	17 (0.34%)	343.9
20	4	M	Sp	11/21/2023	Adv	DFA	Adv B3 (100%)<br>AAV A2 (99.98%)	OR487155.1<br>OP161118.1	Adv (+)<br>AAV (+)	454364	17360 (3.82%)	83 (0.02%)	162600 (0.02%)	399	435139	10312 (2.37%)	792 (0.18%)	1853 (0.18%)	334
24	3	M	Sp	11/28/2023	Adv	DFA	Adv B3 (98.60%)	LC851179.1	Adv (+)	178015	139334 (78.27%)	272 (0.15%)	8085 (4.54%)	202.9	86714	2787 (3.21%)	89 (0.10%)	11 (0.01%)	207.5
25	12	F	Sp	11/28/2023	Adv	DFA	Adv B14 (99.97%)	JN032132.1	Adv (+)	93285	1815 (1.95%)	247 (0.26%)	22958 (24.61%)	313.5	86593	1540 (1.78%)	374 (0.43%)	4 (0.00%)	206
26	0	F	Sp	11/27/2023	RSV	DFA	RSV A (5.17%)	PP525326.1	RSV (+)	148542	121778 (81.98%)	37 (0.02%)	4 (0.00%)	143.5	69932	326 (0.47%)	108 (0.15%)	64 (0.09%)	195.4
27	3	F	Sp	11/27/2023	Adv	DFA	Adv B3 (98.31%)	MK813914.1	Adv (+)	70093	18441 (26.31%)	13 (0.02%)	12944 (18.47%)	274.4	43245	758 (1.75%)	306 (0.71%)	6 (0.01%)	216.9
28	0	F	Sp	11/27/2023	RSV	DFA	–	–	RSV (+)	124883	1821 (1.46%)	37 (0.03%)	14 (0.01%)	222.7	105253	311 (0.30%)	564 (0.54%)	6 (0.01%)	221.4
29	2	M	Sp	11/30/2023	RSV	DFA	RSV A (43.91%)	PQ117647.1	RSV (+)	88895	486 (0.55%)	13 (0.01%)	N/D	238.4	106766	585 (0.55%)	100 (0.09%)	495 (0.46%)	209.6
31	76	F	CSF	12/1/2023	HSV 2	FilmArray	Adv B11 (0%)	AY163756.1	HSV-2 (-)<br>Adv (+)	15635	53 (0.34%)	28 (0.18%)	1 (0.01%)	207.5	9052	64 (0.71%)	63 (0.70%)	2 (0.02%)	215.5
32	9	M	Sp	12/4/2023	Adv	FilmArray	Adv B (0%)	NC_011202.1	Adv (+)	14498	1026 (7.08%)	41 (0.28%)	2 (0.01%)	212.6	5848	384 (6.57%)	35 (0.60%)	2 (0.03%)	204.1
33	3	F	NA	12/4/2023	Adv	DFA	Adv B3 (99.56%)	LC851185.1	Adv (+)	11649	1156 (9.92%)	5 (0.04%)	4017 (34.48%)	332.5	7756	1998 (25.76%)	50 (0.64%)	17 (0.22%)	184.5
37	4	M	NA	12/11/2023	Adv	DFA	Adv B3 (99.90%)	LC851185.1	Adv (+)	19357	210 (1.08%)	12 (0.06%)	9094 (46.98%)	379.3	7299	187 (2.56%)	19 (0.26%)	14 (0.19%)	202.9
38	3	F	NA	12/12/2023	PIV 3	DFA	PIV 3 (0.43%)<br>Adv C1 (0%)	OR728657.1<br>AC_000017.1	PIV3 (+)<br>Adv (+)	7172	2892 (40.32%)	9 (0.13%)	3 (0.04%)	205.4	6050	1376 (22.74%)	60 (0.99%)	98 (1.62%)	197
39	2	F	NA	12/18/2023	RSV	DFA	RSV A (12.28%)<br>Adv B11 (0%)	PP525326.1<br>AY163756.1	RSV (+)<br>Adv (+)	14005	10834 (77.36%)	1 (0.01%)	108 (0.77%)	127.3	11302	6966 (61.64%)	1 (0.01%)	274 (2.42%)	134.8
40	1	F	NA	12/18/2023	Adv	DFA	Adv B3 (99.93%)	MK813914.1	Adv (+)	11306	195 (1.72%)	7 (0.06%)	5206 (46.05%)	348.5	6447	819 (12.70%)	28 (0.43%)	12 (0.19%)	178.8
41	5	M	NA	12/18/2023	Adv	DFA	Adv B3 (99.37%)	LC851185.1	Adv (+)	20443	10036 (49.09%)	2 (0.01%)	3594 (17.58%)	283.3	18926	11881 (62.78%)	22 (0.12%)	79 (0.42%)	141.6
42	7	F	NA	12/18/2023	Adv	DFA	Adv B3 (99.76%)	LC851189.1	Adv (+)	17487	126 (0.72%)	5 (0.03%)	9209 (52.66%)	363.6	19239	1808 (9.40%)	262 (1.36%)	42 (0.22%)	205.4
43	4	M	NA	12/19/2023	Adv	DFA	Adv B3 (56.13%)	LC799991.1	Adv (+)	4711	32 (0.68%)	3 (0.06%)	1307 (27.74%)	231	12145	2745 (22.60%)	80 (0.66%)	15 (0.12%)	192.8
44	1	M	NA	12/20/2023	PIV 3	DFA	–	–	PIV3 (+)	9929	349 (3.51%)	29 (0.29%)	1 (0.01%)	208.1	15298	1217 (7.96%)	53 (0.35%)	7 (0.05%)	210.1
45	9	F	NA	12/20/2023	PIV 2	DFA	PIV 2 (1.10%)	NC_003443.1	PIV2 (+)	5738	159 (2.77%)	6 (0.10%)	1 (0.02%)	207.3	24387	5201 (21.33%)	122 (0.50%)	14 (0.06%)	207.5
46	3	M	NA	12/20/2023	Adv	DFA	Adv B3 (0%)	OQ518278.1	Adv (+)	15944	7726 (48.46%)	31 (0.19%)	267 (1.67%)	205	26056	18665 (71.63%)	13 (0.05%)	4 (0.02%)	117.4
47	3	M	NA	12/20/2023	PIV 3	DFA	PIV 3 (0%)	MF973173.1	PIV3 (+)	11120	530 (4.77%)	17 (0.15%)	853 (7.67%)	236	11151	3470 (31.12%)	86 (0.77%)	60 (0.54%)	188.4
48	3	M	NA	12/20/2023	Adv	DFA	Adv B3 (99.25%)	LC703523.1	Adv (+)	10589	2170 (20.49%)	8 (0.08%)	3602 (34.02%)	310	14111	4355 (30.86%)	93 (0.66%)	119 (0.84%)	192.1
49	1	M	NA	12/20/2023	Adv	DFA	Adv C2 (80.52%)	MH828485.1	Adv (+)	19544	2607 (13.34%)	28 (0.14%)	2940 (15.04%)	285.4	10224	2929 (28.65%)	117 (1.14%)	30 (0.29%)	181.9
50	4	M	NA	12/20/2023	Adv	DFA	Adv B3 (99.78%)	MK813914.1	Adv (+)	13625	577 (4.23%)	2 (0.01%)	5557 (40.79%)	303.2	7814	4724 (60.46%)	16 (0.20%)	31 (0.40%)	134.7
51	11	M	NA	12/21/2023	Adv	DFA	Adv B3 (87.28%)	MK813914.1	Adv (+)	11802	4938 (41.84%)	1 (0.01%)	2218 (18.79%)	222.5	10428	5150 (49.39%)	38 (0.36%)	14 (0.13%)	172.1
52	7	M	NA	12/25/2023	Adv	DFA	Adv B11 (0%)	AY163756.1	Adv (+)	15169	9771 (64.41%)	NA	131 (0.86%)	115.2	14380	8511 (59.19%)	15 (0.10%)	9 (0.06%)	118.6
53	7	F	NA	12/25/2023	Adv	DFA	Adv B3 (3.09%)	OQ518260.1	Adv (+)	23365	13417 (57.42%)	22 (0.09%)	500 (2.14%)	178.6	9166	5821 (63.51%)	3 (0.03%)	3 (0.03%)	108.9
54	2	F	NA	12/25/2023	HRV<br>PIV 3	FilmArray<br>FilmArray	HRV (0%)<br>–	PP194074.1<br>–	HRV (+)<br>PIV3 (-)	21713	9166 (42.21%)	63 (0.29%)	5 (0.02%)	209.3	13716	8877 (64.72%)	26 (0.19%)	N/D	86.4
55	5	M	NA	12/25/2023	Adv	DFA	Adv B11 (0%)	AY163756.1	Adv (-)	6285	229 (3.64%)	8 (0.13%)	34 (0.54%)	197.6	4206	1329 (31.60%)	17 (0.40%)	1 (0.02%)	162.7
56	1	F	NA	12/26/2023	Adv	DFA	Adv C1 (0%)	AC_000017.1	Adv (+)	25655	14310 (55.78%)	103 (0.40%)	62 (0.24%)	106.5	9613	392 (4.08%)	75 (0.78%)	8 (0.08%)	182.5
57	0	F	NA	12/26/2023	PIV 3	DFA	PIV 3 (13.37%)	PP419962.1	PIV3 (+)	25921	15204 (58.66%)	105 (0.41%)	10 (0.04%)	113.2	41861	12658 (30.24%)	4888 (11.68%)	126 (0.30%)	166
58	4	M	NA	12/27/2023	Adv	DFA	Adv B3 (87.74%)	OR487155.1	Adv (+)	18828	6307 (33.50%)	4 (0.02%)	2463 (13.08%)	166	18817	3828 (20.34%)	681 (3.62%)	16 (0.09%)	165.8
59	6	F	NA	12/27/2023	Adv	DFA	Adv B11 (0%)	AY163756.1	Adv (+)	12397	4082 (32.93%)	32 (0.26%)	124 (1.00%)	133.4	12768	588 (4.61%)	89 (0.70%)	N/D	173.2
60	6	M	NA	12/29/2023	Adv	DFA	Adv B3 (1.31%)	MK813914.1	Adv (+)	28464	16151 (56.74%)	10 (0.04%)	374 (1.31%)	95.4	12282	278 (2.26%)	141 (1.15%)	2 (0.02%)	157.4
61	3	F	NA	12/29/2023	Adv	DFA	Adv B3 (98.54%)	MK813914.1	Adv (+)	26924	1238 (4.60%)	1 (0.00%)	10684 (39.68%)	293.5	31543	6052 (19.19%)	302 (0.96%)	11 (0.03%)	168.4
62	6	F	NA	12/29/2023	Adv	DFA	Adv B3 (96.54%)	MK813914.1	Adv (+)	30863	10313 (33.42%)	29 (0.09%)	4555 (14.76%)	239	17434	1158 (6.64%)	136 (0.78%)	N/D	159.6

63	4	F	NA	1/2/2024	Adv	DFA	Adv B (0%)	EF011630.1	Adv (+)	15362	7104 (46.24%)	1 (0.01%)	141 (0.92%)	95.3	23336	12898 (55.27%)	100 (0.43%)	2 (0.01%)	87.8
64	4	M	NA	1/2/2024	Adv	DFA	Adv B3 (41.02%)	MK813914.1	Adv (+)	27103	7318 (27.00%)	40 (0.15%)	1212 (4.47%)	132.1	25683	20 (0.08%)	11573 (45.06%)	N/D	161.7
65	6	M	NA	1/2/2024	Adv	DFA	Adv B3 (98.86%)	MK813914.1	Adv (+)	36538	142 (0.39%)	6 (0.02%)	16657 (45.59%)	329.1	19622	961 (4.90%)	93 (0.47%)	3 (0.02%)	191.7
66	4	M	NA	1/2/2024	Adv	DFA	Adv B3 (98.30%)	MK813914.1	Adv (+)	34342	11758 (34.24%)	3 (0.01%)	7105 (20.69%)	236.1	24300	3841 (15.81%)	942 (3.88%)	22 (0.09%)	184
67	6	F	NA	1/2/2024	Adv	DFA	Adv B3 (98.54%)	MK813914.1	Adv (+)	28384	744 (2.62%)	22 (0.08%)	12157 (42.83%)	334.8	30773	26068 (84.71%)	68 (0.22%)	1 (0.00%)	96.4
68	7	F	NA	1/4/2024	Adv	DFA	Adv B3 (98.56%)	MK813914.1	Adv (+)	27690	3282 (11.85%)	1 (0.00%)	10042 (36.27%)	297.6	32985	15066 (45.68%)	98 (0.30%)	2 (0.01%)	143.5
69	4	M	NA	1/4/2024	Adv	DFA	Adv B3 (90.79%)	MK813914.1	Adv (+)	44274	26050 (58.84%)	6 (0.01%)	2461 (5.56%)	153.1	24567	3914 (15.93%)	154 (0.63%)	4 (0.02%)	185.3
70	17	F	NA	1/4/2024	IAV	DFA	IAV (H3N2)<br>(Seg 1: 88.17%)<br>(Seg 2: 85.52%)<br>(Seg 3: 79.31%)<br>(Seg 4: 88.59%)<br>(Seg 5: 59.41%)<br>(Seg 6: 88.87%)<br>(Seg 7: 49.95%)<br>(Seg 8: 36.03%)	PQ068011.1<br>PQ632956.1<br>OY998358.1<br>OY998351.1<br>PQ068512.1<br>OY998353.1<br>PP535505.1<br>PP535516.1	IAV (+)	23716	6904 (29.11%)	29 (0.12%)	4 (0.02%)	165.5	30263	1274 (4.21%)	145 (0.48%)	353 (1.17%)	218.2
71	7	M	NA	1/5/2024	Adv	DFA	Adv B3 (19.13%)	LC799991.1	Adv (+)	41387	28646 (69.21%)	2 (0.00%)	934 (2.26%)	126	31629	18444 (58.31%)	76 (0.24%)	8 (0.03%)	165.9
72	2	F	NA	1/5/2024	Adv	DFA	Adv B3 (92.16%)	MK813914.1	Adv (+)	40336	22812 (56.55%)	5 (0.01%)	3068 (7.61%)	178.9	22395	3283 (14.66%)	72 (0.32%)	9 (0.04%)	167.9
73	3	M	NA	1/8/2024	Adv	DFA	Adv B3 (1.29%)	OQ518267.1	Adv (+)	20292	10711 (52.78%)	5 (0.02%)	440 (2.17%)	90.2	9356	446 (4.77%)	64 (0.68%)	5 (0.05%)	152.9
74	2	F	NA	1/8/2024	Adv	DFA	Adv B3 (96.41%)	OQ518278.1	Adv (+)	15667	505 (3.22%)	2 (0.01%)	7763 (49.55%)	321.2	15409	3935 (25.54%)	111 (0.72%)	14 (0.09%)	140.5
75	6	F	NA	1/8/2024	Adv	DFA	Adv B3 (88.16%)	MK813914.1	Adv (+)	21170	9291 (43.89%)	2 (0.01%)	2071 (9.78%)	148.7	11845	1224 (10.33%)	413 (3.49%)	2 (0.02%)	175
76	6	M	NA	1/9/2024	Adv	DFA	Adv B3 (61.76%)	MK813914.1	Adv (+)	10753	608 (5.65%)	29 (0.27%)	1238 (11.51%)	184.2	15997	327 (2.04%)	232 (1.45%)	5 (0.03%)	187.9
77	6	M	NA	1/9/2024	Adv	DFA	Adv B3 (98.81%)	OR487155.1	Adv (+)	21259	312 (1.47%)	48 (0.23%)	3806 (17.90%)	235.9	21024	1177 (5.60%)	172 (0.82%)	5 (0.02%)	193.5
78	3	F	NA	1/10/2024	IAV	DFA	IAV<br>(Seg 1:.0%)<br>(Seg 2: 0%)<br>(Seg 3: 0%)<br>(Seg 4: 0%)<br>(Seg.5: 0%)<br>(Seg 6: 0%)<br>(Seg 7: 0%)<br>(Seg 8: 0%)	NC_007373.1<br>NC_007372.1<br>NC_007371.1<br>NC_007366.1<br>NC_007369.1<br>NC_007368.1<br>NC_007367.1<br>NC_007370.1	IAV (-)	32014	19711 (61.57%)	25 (0.08%)	N/D	112.3	19613	2254 (11.49%)	239 (1.22%)	8 (0.04%)	192
79	4	M	NA	1/11/2024	PIV3<br>HRV<br>EV	FilmArray<br>FilmArray<br>FilmArray	PIV 3 (99.64%)<br>–<br>–	MN145875.1<br>–<br>–	PIV3 (+)<br>HRV (-)<br>EV (-)	32094	13308 (41.47%)	50 (0.16%)	5 (0.02%)	172.5	26149	6642 (25.40%)	164 (0.63%)	1818 (6.95%)	229.3
80	1	F	NA	1/12/2024	Adv	DFA	Adv B3 (99.92%)	MK813914.1	Adv (+)	24387	3614 (14.82%)	18 (0.07%)	9754 (40.00%)	349.7	20685	3993 (19.30%)	211 (1.02%)	7 (0.03%)	172.2
81	9	M	NA	1/15/2024	Adv	DFA	Adv B11 (0%)	AY598970.1	Adv (+)	19183	11420 (59.53%)	ND	264 (1.38%)	94.6	19360	1653 (8.54%)	286 (1.48%)	5 (0.03%)	203.6
82	5	F	NA	1/15/2024	Adv	DFA	Adv B3 (99.50%)	LC851185.1	Adv (+)	26469	7071 (26.71%)	7 (0.03%)	9032 (34.12%)	298.7	19362	7705 (39.79%)	149 (0.77%)	97 (0.50%)	146.1
83	9	M	NA	1/15/2024	Adv	DFA	Adv B3 (99.94%)	LC799991.1	Adv (+)	30226	677 (2.24%)	5 (0.02%)	15784 (52.22%)	360.4	22416	3683 (16.43%)	479 (2.14%)	11 (0.05%)	162
84	7	M	NA	1/15/2024	Adv	DFA	Adv B3 (17.77%)	MK813914.1	Adv (+)	15666	10478 (66.88%)	3 (0.02%)	825 (5.27%)	149.2	24435	4111 (16.82%)	286 (1.17%)	9 (0.04%)	185.1
86	2	M	NA	1/16/2024	Adv	DFA	Adv C5 (10.24%)	OR777181.1	Adv (+)	27750	14280 (51.46%)	40 (0.14%)	735 (2.65%)	142	33364	7635 (22.88%)	212 (0.64%)	1 (0.00%)	196.3
87	5	M	NA	1/16/2024	Adv	DFA	Adv B3 (99.41%)	LC851189.1	Adv (+)	25310	3944 (15.58%)	65 (0.26%)	3604 (14.24%)	232.8	17115	1628 (9.51%)	228 (1.33%)	1 (0.01%)	181.5
88	3	M	NA	1/17/2024	CoV HKU1<br>Adv	FilmArray<br>FilmArray	CoV HKU1 (0%)<br>–	LC654448.1<br>–	CoV-HKU1 (-)<br>Adv (+)	7363	1132 (15.37%)	20 (0.27%)	4 (0.05%)	165.4	34932	18800 (53.82%)	229 (0.66%)	53 (0.15%)	186.5
89	7	F	NA	1/18/2024	Adv	DFA	Adv B3 (98.56%)	MK813914.1	Adv (+)	13260	822 (6.20%)	2 (0.02%)	5845 (44.08%)	301.9	15484	5933 (38.32%)	45 (0.29%)	17 (0.11%)	87.4
90	3	F	NA	1/19/2024	Adv	DFA	Adv B3 (99.95%)	MK813914.1	Adv (+)	34870	2813 (8.07%)	58 (0.17%)	9587 (27.49%)	297.9	27165	2235 (8.23%)	323 (1.19%)	2 (0.01%)	202.8
91	5	M	Sp	1/22/2024	EV<br>PIV2<br>HRV	FilmArray<br>FilmArray<br>FilmArray	EV A (61.42%)<br>PIV2 (5.60%)<br>–	NC_001612.1<br>NC_003443.1<br>–	EV (+)<br>PIV2 (+)<br>HRV (+)	19157	13289 (69.37%)	3 (0.02%)	2 (0.01%)	67	26276	9859 (37.52%)	191 (0.73%)	13 (0.05%)	186.9
92	8	M	NA	1/22/2024	Adv	DFA	Adv B3 (25.28%)	LC799991.1	Adv (+)	17943	9107 (50.76%)	15 (0.08%)	1000 (5.57%)	142.6	32993	8424 (25.53%)	274 (0.83%)	10 (0.03%)	181.3
93	7	F	NA	1/22/2024	Adv	DFA	Adv B3 (60.40%)	MK813914.1	Adv (+)	12742	5000 (39.24%)	15 (0.12%)	1320 (10.36%)	186.1	22325	7006 (31.38%)	160 (0.72%)	5 (0.02%)	177.5
94	6	F	NA	1/22/2024	Adv	DFA	Adv B3 (99.25%)	LC851179.1	Adv (+)	13180	2825 (21.43%)	3 (0.02%)	4099 (31.10%)	267	23124	8818 (38.13%)	271 (1.17%)	65 (0.28%)	179.5

1.**Specimen Type:** St, stool; Sp, sputum; CSF, cerebrospinal fluid; NA, nasopharyngeal aspirate.

2.**Clinical ID / NGS ID / Research Lab PCR Test:** Adv: Human Adenovirus; AAV: Adeno-associated virus; CoV: Coronavirus; EV: Enterovirus; IAV: Inflenza A virus; ICV: Inflenza C virus; HRV: Human Rhinovirus; PIV: Human parainfluenza virus; RSV: Respiratory Syncytial Virus; SaV: Sapporovirus

3.**Clinical Test Method:** DFA: Direct Fluorescent Assay; CLIA: Chemiluminescent Immunoassay

4.**NGS read composition (Total/Host/Bacteria/Virus Reads):** N/D, not detected (no reads identified for that category). Virus reads include all viral sequences detected, not limited to human viruses (may also include plant viruses or bacteriophages).
