# Supplementary material for: Multiplex metagenomic sequencing for rapid viral pathogen identification and surveillance in clinical specimens
Source: BMC Infect Dis. 2025 Nov 10;25:1531. doi: 10.1186/s12879-025-11952-w (PMC12604265; doi:10.1186/s12879-025-11952-w)
Supplement: Supplementary file 5 — Supplementary Material 5 [file 12879_2025_11952_MOESM5_ESM.pdf]

Supplementary Table S2. Primer list for conventional PCR.

Target	Sequences
Adenovirus*	F': 5'-GGA CGC CTC GGA GTA CCT GAG-3'
	R': 5'-ACI GTG GGG TTT CTG AAC TTG TT-3'
Picornaviridae (Enterovirus and Rhinovirus)	F'.:5'-AAG CAC TTC TGT TTC C-3'
	R': 5'-CAT TCA GGG GCC GGA GGA-3'
Rhinovirus*	F: 5'-GGC CCC TGA ATG YGG CTA A-3'
	R: 5'-GAA ACA CGG ACA CCC AAA GTA G-3'
Coronavirus*	F': 5'-ACW CAR HTV AAY YTN AAR TAY GC-3'
	R': 5'-TCR CAY TTD GGR TAR TCC CA-3'
Herpesvirus (HSV-1, HSV-2, CMV and EBV)	F': 5'-GAC TTT GCC AGC CTG TAC C-3'
	R': 5'-GTC CGT GTC CCC GTA GAT G-3'
Human respiratory syncytial virus (Type A)*	F': 5'-ACT GCA ATC AYA CAA GAT GCA ACR A-3'
	R': 5'-CAG ATT GRA GAA GCT GAT TCC A-3'
Human respiratory syncytial virus (Type B)*	F': 5'-ACT TAC CTT ACT CAA GTC TCA CCA GAA A-3'
	R': 5-TTG TRG CTG ART TTG TGT GGA T-3’
Influenza A virus*	F’: 5'-GAC CRA TCC TGT CAC CTC TGA C-3'
	R’: 5'-AGG GCA TTY TGG ACA AAK CGT CTA-3'
Influenza C virus	F’: 5'-GCT CCA AGC AAC ATA GCA CC-3'
	R’: 5'-TCG GTA GCC ATA ACG AAT CC-3'
Adeno-associated virus	F’: 5'-AAC TGG ACC AAT TGA AAA CTT TCC-3'
	R’: 5'-AAA AAG TCT TTG ACT TCC TGC TT-3'
Sapporo virus*	F’: 5'-GAY YWG GCY CTC GCY ACC TAC-3'
	R’: 5'-CCC BGG TGG KAY GAC AGA AG-3'

* Degenerate bases are indicated using IUPAC nucleotide codes: I = Inosine; Y = C or T; W = A or T; R = A or G; H = A or C or T; V = A or C or G; D = A or G or T.
